# Influence of Platelet Lysate on 2D and 3D Amniotic Mesenchymal Stem Cell Cultures

**DOI:** 10.3389/fbioe.2019.00338

**Published:** 2019-11-15

**Authors:** Markus Pasztorek, Eva Rossmanith, Christoph Mayr, Fabian Hauser, Jaroslaw Jacak, Andreas Ebner, Viktoria Weber, Michael B. Fischer

**Affiliations:** ^1^Department for Biomedical Research, Center of Experimental Medicine, Danube University Krems, Krems an der Donau, Austria; ^2^Department of Applied Experimental Biophysics, Institute of Biophysics, Johannes Kepler University Linz, Linz, Austria; ^3^School of Medical Engineering and Applied Social Sciences, University of Applied Sciences Upper Austria, Linz, Austria; ^4^Christian Doppler Laboratories, Department for Biomedical Research, Danube University Krems, Krems an der Donau, Austria

**Keywords:** mesenchymal stem cells, stress fibers, mitochondrial dynamics, platelet lysate, membrane elasticity

## Abstract

The mechanobiological behavior of mesenchymal stem cells (MSCs) in two- (2D) or three-dimensional (3D) cultures relies on the formation of actin filaments which occur as stress fibers and depends on mitochondrial dynamics involving vimentin intermediate filaments. Here we investigate whether human platelet lysate (HPL), that can potentially replace fetal bovine serum for clinical-scale expansion of functional cells, can modulate the stress fiber formation, alter mitochondrial morphology, change membrane elasticity and modulate immune regulatory molecules IDO and GARP in amnion derived MSCs. We can provide evidence that culture supplementation with HPL led to a reduction of stress fiber formation in 2D cultured MSCs compared to a conventional growth medium (MSCGM). 3D MSC cultures, in contrast, showed decreased actin concentrations independent of HPL supplementation. When stress fibers were further segregated by their binding to focal adhesions, a reduction in ventral stress fibers was observed in response to HPL in 2D cultured MSCs, while the length of the individual ventral stress fibers increased. Dorsal stress fibers or transverse arcs were not affected. Interestingly, ventral stress fiber formation did not correlate with membrane elasticity. 2D cultured MSCs did not show differences in the Young's modulus when propagated in the presence of HPL and further cultivation to passage 3 also had no effect on membrane elasticity. In addition, HPL reduced the mitochondrial mass of 2D cultured MSCs while the mitochondrial mass in 3D cultured MSCs was low initially. When mitochondria were segregated into punctuate, rods and networks, a cultivation-induced increase in punctuate and network mitochondria was observed in 2D cultured MSCs of passage 3. Finally, mRNA and protein expression of the immunomodulatory molecule IDO relied on stimulation of 2D culture MSCs with pro-inflammatory cytokines IFN-γ and TNF-α with no effect upon HPL supplementation. GARP mRNA and surface expression was constitutively expressed and did not respond to HPL supplementation or stimulation with IFN-γ and TNF-α. In conclusion, we can say that MSCs cultivated in 2D and 3D are sensitive to medium supplementation with HPL with changes in actin filament formation, mitochondrial dynamics and membrane elasticity that can have an impact on the immunomodulatory function of MSCs.

## Introduction

For clinical-scale expansion of functional mesenchymal stem cells (MSCs) the use of xeno-based serum products are prohibited, but human platelet lysate (HPL) can potentially replace fetal bovine serum (FBS) the most widely used medium supplement in the past (Henschler et al., 2019). According to the European Medicines Agency and the European Commission (regulation number 1394/2007), MSCs belong to advanced-therapy medicinal products (ATMPs) that need to be produced under good manufacturing practice (GMP) conditions. FBS was banned because xeno-proteins can be taken up by MSCs and remained internalized during culture with a peri-nuclear localization (Spees et al., 2004) and patients developed antibodies against bovine antigens after receiving cell transplantation with MSCs expanded in FBS containing medium (Antoninus et al., 2015). Certain FBS lots, in addition, possess endotoxin content and can be a source of microbial contaminations, including viruses, bacteria, fungi, and prions. Therefore, human-derived FBS alternatives were developed such as HPLs and thrombin-induced or TRAP-induced platelet releasate (secretome) but MSCs were shown to be sensitive to medium supplementation during expansion, which can result in distinct supportive signaling by MSCs in processes of tissue regeneration or immune modulation.

An important issue for cultivation and expansion of MSCs, next to the culture medium selection, is the mechanobiological effect of 2D cultivation (the adherence of MSCs to coated plastic surfaces) or 3D cultivation (MSCs grown in spherical aggregate where cells produce autologous matrix components) (Dominici et al., 2006; Zhou et al., 2017). Depend on 2D or 3D cultivation, MSCs assemble actin filaments differently, which occur as branched stress fibers (SF) (Tojkander et al., 2012; Burridge and Wittchen, 2013; Burridge and Guilluy, 2016). They can be segregated into ventral and dorsal SFs or transverse arcs according to their appearance within the cell and their coupling to integrin-containing focal adhesions (FAs) (Tojkander et al., 2012; Xia and Kanchanawong, 2017). Ventral SFs are typically located at the ventral surface of the cell anchored at both ends to FAs, are several micrometers long and span the entire length of the cell. Dorsal SFs, in contrast, are usually shorter, appear radial to the cell and anchored at just one end to a FA. Transverse arcs are not bound to FAs at all. FAs are multi-protein subcellular structures comprising of talin, alpha-actinin, filamin, vinculin, paxillin, focal adhesion kinase, and tensin linking actin filaments to integrins on the cell surface to facilitate adhesion (Atherton et al., 2016). Proteins of FAs are in a continuous flux within the cytoplasm of MSCs with proteins constantly associating and dissociating in particular during cells in motion (Holle et al., 2013; Bays et al., 2014). Under static conditions, cell-generated forces can be transmitted across integrin-containing FAs to the surrounding extracellular matrix (ECM) network (Holle et al., 2013; Bays et al., 2014). In a reciprocal manner the ECM can influence actin filament formation, thereby controlling cell shape, regulate the balance between cell growth, differentiation and death (Zeiger et al., 2012; Yang et al., 2016). Considerations on the effect of the cell-matrix environment can help to solve the performance gap between physiological stem cell niches and engineered artificial environments for specific applications ranging from basic science to stem cell therapies (Gattazzo et al., 2014; Mullen et al., 2014; LeBlon et al., 2015).

Due to the potential of MSC application in regenerative medicine, the role of mitochondrial metabolism during cultivation in different culture media, have received substantial attention from life scientists and clinicians (Hsu et al., 2016). Intracellular organelles, including mitochondria are mechanoresponsive and undergo dynamic remodeling during MSC cultivation (Jackson and Krasnodembskaya, 2017). Mitochondria are the powerhouse of the cell and besides energy generation, they participate in calcium signaling, redox homeostasis and apoptosis (Rodriguez et al., 2018; Li C. et al., 2019). Mitochondrial accumulation at sites of high-energy demand, within the cytoplasm, is required to achieve local ATP production essential for virtually all cellular functions. Furthermore, stationary mitochondria serve as calcium buffers to avoid harmful intracellular calcium peaks. Mitochondrial fusion and fission dynamics involve vimentin intermediate filaments (IF). Binding of mitochondria to vimentin IFs is tightly regulated by GTPase Rac1 that acts through its effector PAK1 kinase and causes the phosphorylation of vimentin on Ser-55 modifying the mitochondria-binding site (Shankar et al., 2013). Consequently, released mitochondria can acquire the higher motility and the reduced mitochondrial membrane potential (MMP). Differences in vimentin IF formation can have influence on MSC function, as the timely delivery and distributions of mitochondria in the cytoplasm is crucial in particular for a fine-tuned mitochondrial fusion and fission process (Gruenbaum and Aebi, 2014; Pérez-Sala et al., 2015). MSCs are reliant on low levels of stress signals in the stem cell niche and intact mitochondrial metabolism for sufficient ATP production to maintain stemness (Zhou et al., 2017). In addition to stemness, the immunomodulatory characteristics of 3D cultured MSCs was enhanced as defined by gene expression profile analysis at mRNA and protein level as compared to 2D cultured MSCs, but a link between immunomodulatory function of MSCs with SF formation, mitochondrial morphology and membrane elasticity was not described in detail (Li et al., 2018).

Here we investigated the actin filament formation and mitochondrial morphology in 2D and 3D MSC cultures by flow cytometry or laser scanning confocal microscopy (LSM) in response to HPL, applied an MSC migration assay, studied force-distance profiles by atomic force microscopy (AFM) and analyzed the expression of immunomodulatory molecules GARP or IDO following pro-inflammatory stimulation with a combination of IFN-γ and TNF-α by RT-PCR and western blotting (Probst-Kepper and Buer, 2010; Shi et al., 2011; Carrillo-Galvez et al., 2015; Pérez-Sala et al., 2015; Niu et al., 2017; Najar et al., 2018; Li H. et al., 2019).

## Materials and Methods

### Isolation, Culturing, Three-Lineage Differentiation, and Migration of MSCs

The study was approved by the ethic commission of the Medical University Vienna (EK791/2008, EK1192/2015), the University Hospital of Lower Austria (GS1-EK-4/312-2015) and the Danube University Krems (Nr. 821/2009). The placenta was obtained from healthy delivering woman in accordance with the Austrian Hospital Act (KAG 1982) after written informed consent was signed. The amnion was separated from the placenta and cut in 3 × 3 cm squares. The slices were washed in physiological NaCl and digested with dispase (2.5 CU/ml, Becton Dickinson, Franklin Lakes, NJ) for 9 min at 37°C (Soncini et al., 2007). The fragments were incubated in a mix of collagenase A (1 mg/ml) and DNase I (0.01 mg/ml, both Roche, Basel, Switzerland) for 2 h at 37°C. For the separation of the cells from the tissue, the mix was centrifuged at 180 g for 5 min and the supernatant strained with a 100 μm cell-strainer. The cell suspension was centrifuged at 360 g for 10 min and amnion cells were adhered to plastic surfaces to propagate MSCs in 2D within MSCGM™- medium (Lonza Group Ltd., Basel, Switzerland) or MSCBM™ with 8% HPL (MacoPharma, Mouvaux, France), supplemented with 100 U/ml penicillin, 100 μg/ml streptomycin and 250 ng/ml amphotericin B (all from Gibco, Thermo Fisher Scientific, Waltham, MA) at 37°C in 5% CO_2_ humidified environment (Stericycle, Thermo Fisher Scientific). Every 3 to 4 days medium was changed and MSCs were passaged at 80% confluence. For each experiment amnion-derived stromal cells were characterized by flow cytometry. Three-lineage differentiation of MSCs into chondrogenic, osteogenic, and adipogenic lineage (Supplementary Figure 2C) was performed using standard protocols (Walzer et al., 2019). For the migration assay, P3 MSCs (30 000 cells/well) were grown in 6 well plates with 2 well biocompatible silicone inserts with a defined 500 μm cell-free gap according to the manufacturer's instructions (Ibidi, Gräfelfing, Germany) and the analysis was performed according to a previous publication (Jonkman et al., 2014).

### Preparation of MSC Spheroids

Adherent amnion derived stromal cells in passage 0 (P0) were grown to 80% confluence and the monolayers rinsed with PBS. Accutase (Gibco, Thermo Fisher Scientific) was added, incubated at 37°C until cells detach and stopped by adding 5 ml of PBS. The cells were centrifuged at 300 g for 5 min and counted. Five thousand cells/25 μl drop were pipetted on a lid from a 100 mm petri dish (Greiner Bio-One, Kremsmünster, Austria) and incubated as hanging drops at 37°C in 5% CO_2_ humidified environment. The aggregate formation was monitored by a stereo microscope (Olympus IMT2) and spheroids were collected after 2 days and forwarded to characterization by flow cytometry.

### Flow Cytometry Analysis of MSCs

Living MSCs (Live/Dead Cell Assay, Invitrogen, Thermo Fisher Scientific) from 2D cultures or spheroids in P1 were characterized for the expression of ecto-5'-nucleotidase (APC CD73), Thy-1 a glycophosphatidyl-inositol (GPI) anchored conserved cell surface protein (FITC CD90), endoglin a component of the receptor complex of TGF-β (PE-Cy7 CD105) and for glycoprotein A repetitions predominant (eFluor660® GARP) mAb (1 μg/ml, all from eBioscience, Thermo Fisher Scientific) on a Gallios 10/3 flow cytometer (Beckman Coulter GmbH, Krefeld, Germany). Actin and mitochondria quantification was performed using a AlexaFluor® 594 labeled phalloidin (1 U/ml) or MitoTracker™ Green FM (100 nM, both from Molecular Probes, Thermo Fisher Scientific) and size measurements were performed with silica beads [1.5 μm (Kisker Biotech, Steinfurt, Germany), 30 and 65 μm (Beckman Coulter GmbH)].

### High Resolution Imaging of MSCs by Scanning Electron Microscopy

Spherical MSC aggregates or a suspension of 1 × 10^6^ MSCs/ml were incubated in 24 well plates (Nunc, Thermo Fisher Scientific) on Nunc™Thermanox™ coverslips (Nunc, Thermo Fisher Scientific) for 16 h at 37°C in 5% CO_2_ humidified environment. After three times washing with PBS, the coverslips were fixed with 2.5% glutaraldehyde (Sigma-Aldrich, St. Louis, MO) for 20 min at 4°C, washed two times again and dehydrated by stepwise alcohol extraction from 50 to 100%. The coverslips were mounted on EM-Tec CT12 conductive double side adhesive carbon tabs (Micro to Nano V.O.F., Haarlem, Netherlands) with a diameter of 12 mm and then surface sputtered with gold using a QuorumTech Q150T ES (Quorum Tech Ltd., Laughton, UK) for better resolution. For the scanning electron micrographs of spherical MSC aggregates and single adherent MSCs a FlexSEM 1,000 scanning electron microscope (Hitachi Ltd. Corp., Tokyo, Japan) with SEM MAP camera in SEM mode was used.

### Stimulation of MSCs With Pro-inflammatory Cytokines

MSCs in P1 in 2D or 3D cultures were stimulated with 50 ng/ml TNF-α and 50 ng/ml 313 IFN-γ (both from PeproTech, Rocky Hill, NJ) in MSCGM™- 314 medium or MSCBM™ with 8% HPL for 24 and 48 h.

### Confocal Laser Scanning Microscopy for Investigating the Cyto-Architecture of Cultured MSCs

MSCs were grown in chamber slides for 72 h or alternatively MSC spherical aggregates were adhered to plastic surface for 16 h. The cells were fixed with fixation and permeabilization reagent (eBioscience, Thermo Fisher Scientific) and incubated either with vinculin (2 μg mouse mAb/ml, clone 7F9, Santa Cruz Biotechnology, Dallas, TX), with paxillin (2 μg mouse mAb/ml, clone B2 Santa Cruz Biotechnology) to reveal FA, with vimentin (3.6 μg mouse mAb/ml, clone 1/9, Dako Products, Agilent, Santa Clara, CA) to label the IFs, or with the MitoTracker™ Red CMX Ros (100 nM, Molecular Probes, Thermo Fisher Scientific) to reveal mitochondria. Chamber slides were incubated with the second ab, a goat-anti-rabbit polyclonal Fab fragment Ab labeled with AF-488 (3 μg/ml, Jackson Laboratories, Bar Harbor, MN) after two times of washing with PBS. For the labeling of f-actin MSCs were counterstained with AF-488 or AF-594 phalloidin (0.1 U/ml, Molecular Probes, Thermo Fisher Scientific) and finally nuclei were stained with DAPI (Sigma-Aldrich). The slides were mounted with Fluoromount-G™ (Southern Biotechnology, Thermo Fisher Scientific) and analyzed with an alpha-Plan-Apochromat 63x objective and a Leica TCS SP8 confocal microscope (Leica Microsystem GmbH, Wetzlar, Germany). Serial dilutions of each primary and secondary antibody were tested to minimize non-specific adsorption, assure separation of the fluorescent signals, and optimize fluorophore concentration to preclude self-quenching.

### TissueGnostics Technology for a Context-Based Actin Evaluation of Cultured MSCs

MSCs were grown, stained and scanned according to the “confocal microscopy” part. The presence of dorsal or ventral SFs as well as transverse arcs was analyzed by using the analysis software StrataQuest (TissueGnostics, Vienna, Austria). Context based quantitative analysis of fluorescence images on an automatic interface segregated dorsal SFs anchored only at one end to FAs, from ventral SFs spanning f-actin filament bundles from two FAs, and from transverse arcs that are not at all anchored to FAs but instead connected to intracellular structures.

### Atomic Force Microscopy Topographical Imaging and Elasticity Mapping of Cultured MSCs

All samples were prepared in ibidi 35 mm μ-dishes (Ibidi). First of all, the μ-dishes were coated with 5 μg/cm^2^ fibronectin (Sigma-Aldrich). The coating was done by covering the surface with a fibronectin solution and incubation for 60 min at 37°C. Thereafter, the remaining solution was discarded. MSCs were put on the fibronectin coated surface and incubated for 24 h at 37°C in 5% CO_2_ air humidified environment and subsequently immobilized on the surface with 4% formaldehyde (Thermo Fisher Scientific). After immobilization the formaldehyde was discarded, and the μ-dish was filled with 1 ml PBS. A 6000 ILM AFM combination (Keysight, Santa Rosa, CA) was used for all measurements. This system consisted of an inverted microscope (Zeiss Observer D1), a motorized AFM stage (Keysight 6000) and the AFM head itself (N9583A model). The implemented camera was a Hamamatsu C11440. Noise cancellation was done using an Accurion Halcyonics Vario active vibration isolating system combined with an Accurion acoustic damping hood. Calculation of the Young's moduli was done with PicoView 1.18.2 software (Keysight). Further image processing (flattening, statistical evaluation, multilayer images) was performed using Gwyddion 2.45 and PicoImage 5.1.1 software (Keysight). The surface topography of immobilized MSCs was investigated using Bruker MSCT cantilever (Bruker AFM probes, Camarillo, CA). Images were taken in PBS and recorded in contact or tapping mode with 0.5 lines per second. For each sample at least three different cells were measured. The elasticity of immobilized MSCs was investigated by using Bruker MSCT cantilever and by performing force volume measurements in PBS. For most measurements a grid of 32 × 32 points was used. On each measurement point one force distance cycle was performed and the Young's modulus was simultaneously calculated using the Hertz model (Hertz, 1882; Sneddon, 1965; Butt and Jaschke, 1995; Haase and Pelling, 2015; Melzak and Toca-Herrera, 2015). The cycles were done by using a scan rate of 20 μm/s and a scan range of 5 to −5 μm. The maximum force was set to 5 V, which yielded a maximum force exerted on the cells of <2 nN. For each sample three cells were measured in total. For the calculation of the Young's moduli the exact spring constant of the cantilever was evaluated using thermal tune method.

### Western Blotting to Semi-quantify Cellular IDO in Stimulated and Unstimulated MSCs

MSCs were lysed with 1% protease phosphatase inhibitor in RIPA extraction buffer and, total protein was measured using a Agilent Protein 230 kit (Agilent Technologies). Protein was loaded on precast 4–12% polyacrylamide Bis-Tris gels in a NuPage MOPS-buffer and SDS-PAGE western blotting was performed using a XCell SureLock™ Mini-Cell and a PowerEase 500 W power supply. For the transfer on nitrocellulose membranes with a 0.2 μm pore size a XCell II blot™ module was used (Invitrogen, Thermo Fisher Scientific). Membranes were subsequently blocked with non-fat dry milk (Biorad, Hercules, CA). A primary anti-IDO Ab (0.2 mg, clone H-11, Santa Cruz Biotechnology) was detected with HRP conjugate and Clarity Max Western ECL blotting substrate (Biorad) and the chemiluminescent signal was recorded with a Chemi-Doc documentation system (Biorad) for semi-quantification. For internal control, purified IDO protein (Bio-Techne, R&D Systems, Minneapolis, MN) was used at a total concentration of 25–200 ng.

### Human Cytokine Array for HPL Characterization

Human Cytokine profiling was performed with a Proteome Profiler Human XL Cytokine Array Kit (R&D Systems, Minneapolis, MN). MSCBM™- medium (Lonza Group Ltd.) with 8% HPL (MacoPharma) and 3 μl Heparin (5,000 IU/ml, Gilvasan Pharma, Vienna, Austria) was used as sample, the procedure was performed according to the manufacturer's instructions. The chemiluminescent signal was recorded with a Chemi-Doc documentation system (Biorad).

### Semi-quantitative RT-PCR Analysis for IDO and GARP Characterization

Total RNA was extracted from MSCs cultivated in 2D and 3D with and without addition of 50 ng/ml TNF-α and 50 ng/ml IFN-γ using an RNeasy Mini Kit (Qiagen, Venlo, Netherlands) according to the manufacturer's manual. RNA quality was assessed, and the quantity was measured by an RNA 6000 Nano assay (Agilent Technologies). RNA was adjusted to the same concentration for each sample after measuring the RNA concentration. Then, RNA samples were used for cDNA synthesis with the High-Capacity cDNA Transcription Kit (Applied Biosystems, Thermo Fisher Scientific) in a 20 μl reaction mixture according to the manufacturer's protocol. Primers (Metabion—International AG, Planegg, Germany) for HPRT (Toegel et al., 2007), PPIA (Li et al., 2015), IDO (fw: GGAGCAGACTACAAGAAT, rev: TAGCAATGAACATCCAGT) and GARP (Carrillo-Galvez et al., 2015), were used for the qRT-PCR reactions. The qRT-PCR reactions were performed with the SensiMix™ SYBR® Hi-ROX Kit (Bioline, London, UK) according to the manufacturer's protocol. PCR amplification and analyzation were investigated with the LightCycler® 96 Instrument (Roche).

### Mitochondrial 3D dSTORM Analysis

Cultivated MSCs were prepared in 8-well Lab-Tek™ chambers (Nunc, Thermo Fisher Scientific) with two different cultivation media. The cytoskeleton buffer with sucrose (CBS) (Symons and Mitchison, 1991) was used to improve the staining quality, to increase resolution of single molecule signals and to promote mitochondrial staining. MSCs were then fixed using 4% paraformaldehyde, permeabilized with 0.5% Triton X-100 and unspecific binding was blocked with 10 mg/ml albumin from chicken egg white (Sigma-Aldrich). Mitochondria were stained with anti-mitochondria mAb conjugated with AlexaFluor® 488 (5 μg/ml, clone 113-1, Merck Millipore, Darmstadt, Germany) for 60 min in CBS with blocking solution. Fluorescence Microscope images were acquired using a modified Olympus IX81 inverted epifluorescence microscope with an oil-immersion objective (UApo N 60x/1.49 NA, Olympus, Vienna, Austria) and additional tube-lens with a magnification of 1.6x. The signals were detected using an Andor iXonEM+ 897 (back-illuminated) EMCCD camera (16 μm pixel size, Andor Technology, Belfast, Northern Ireland). This results in an image pixel size of 166.667 nm/pixel and a total magnification of 96x. Fluorescence-labeled samples were excited using a 488 nm solid-state laser (diode-pumped, iBeam Smart, Toptica Photonics, Gräfelfing, Germany) and under certain conditions, fluorophores were additional recovered from dark state with a 405 nm diode laser (iPulse, Toptica Photonics). An additional cylindrical lens (f = 500 mm, Thorlabs, Newton, NJ) was introduced in the pathway between camera and the microscope's side port for 3D Super-Resolution microscopy. The fluorescent emission was additionally filtered by a 525/50 nm emission bandpass filter (AHF, Tübingen, Germany). For optimized photo-switching of the rhodamine dye AlexaFluor® 488 a buffer containing EC-Oxyrase® (Sigma-Aldrich), 60% LD-Lactate, 50 mM cystermine and PBS as described (Nahidiazar et al., 2016) has been used. For 3D STORM experiment, a ROI of about 256 × 256 pixels was chosen (depending on cell size) and between 10,000 and 20,000 frames were acquired. The fluorophores were illuminated with 20–30 ms at each frame and an optional 20 ms UV illumination during the readout time of the CCD could be added. Each frames were then analyzed using a custom written analysis software (3D dSTORM-Tools) (Mayr et al., 2018; Sage et al., 2019) and all fitted single molecules were used to reconstruct a 3D Super-Resolution image. Further analysis is based on the 3D point cloud data of the analyzed single molecules. Prior to each experiment, a calibration for the single molecule axial position localization, compensating the axial point spread function distortion, was performed using TetraSpeck (Thermo Fisher Scientific) beads.

### Statistical Analysis

Data were expressed as mean ± standard deviation unless otherwise stated. Mann-Whitney test or unpaired *t*-test were performed for unpaired analysis and paired *t*-test or Wilcoxon test for paired analysis like described in the figure legend. Statistical analysis was performed by the GraphPad Prism software 8.

## Results

### Effect of HPL on MSCs Cultured in Adherent to Plastic Surfaces or in Spheroids

Three batches of HPL from MacoPharma, used for all experiments, were analyzed with a Proteome Profiler Human XL Cytokine Array with 102 cytokines and results are given in a heat map (Supplementary Figure 1A). The analysis of the batches showed largely the same results with certain variations in individual cytokines.

**Figure 1:**
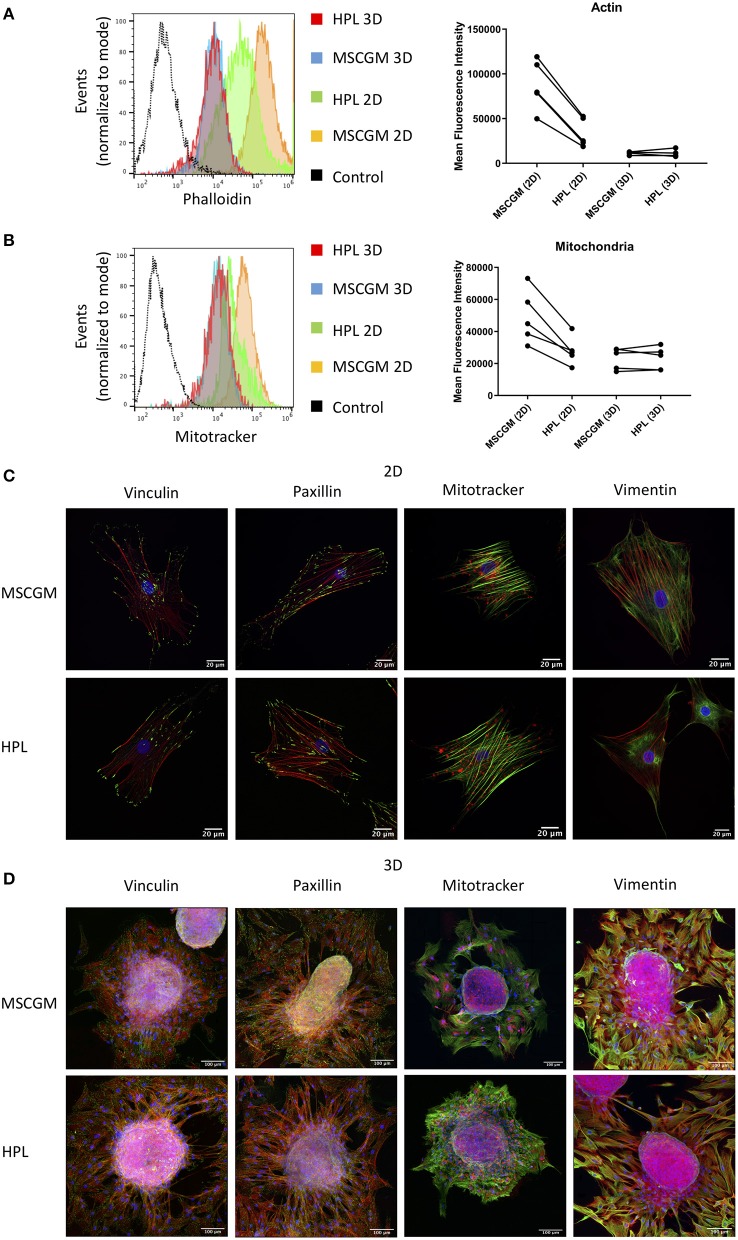
Effect of HPL on 2D and 3D cultures. **(A)** Actin measurements with phalloidin of living CD90^+^ MSCs in 2D vs. 3D cultured in MSCGM™ or MSCBM™ with 8% HPL determined by flow cytometry. Dot plot of the mean fluorescence intensity of four different batches for 3D MSCs and five batches for 2D MSCs of 4,000 cells per batch, one representative flow cytometry histogram is shown. **(B)** Mitochondria measurements with MitoTracker™ of living CD73^+^ MSCs in 2D vs. 3D cultured in MSCGM™ or MSCBM™ with 8% HPL determined by flow cytometry. Dot plot of the mean fluorescence intensity of five different batches, 2,000 MSCs were investigated per batch, one representative flow cytometry histogram is shown. **(C)** Images of passage 1 MSCs cultured in MSCGM™ or MSCBM™ with 8% HPL stained with the specific focal adhesion proteins vinculin and paxillin in green as well as stress fibers (f-actin) in red and nuclei in blue. Mitochondria were stained with MitoTracker™ in red, stress fibers in green and nuclei in blue. Type-3 intermediate filaments are given by staining vimentin in green, stress fibers in red and nuclei in blue. **(D)** Images of MSC spheroids, the staining corresponds to those described in **(A)**. The entire MSC spheroid is a maximal intensity projection of a stitched z-stack 3-channel overlay, magnification was 63x.

Amnion derived MSCs propagated in adherence to plastic surface and analyzed in passage (P)1 or MSCs from spherical aggregates were positive for MSC specific markers CD73, CD90, and CD105 when cultivated in MSCGM™- medium or MSCBM™ with 8% HPL, and showed a size of ~30–60 μm in diameter (Supplementary Figures 2A,B) compared to silica beads with similar refraction index. Only adherent P1 MSCs cultivated in 8% HPL showed a reduction in size (Supplementary Figure 2B). Images from scanning electron microscopy were provided to show that there is no morphological difference between MSCs grown in adherence to plastic surface or MSCs from spherical aggregates cultured with or without HPL (Supplementary Figure 1B). Furthermore, actin filament formation and mitochondrial mass determined by flow cytometry showed that culture supplementation with HPL could reduce the amount of f-actin in adherent P1 MSCs, while this effect could not be observed in MSCs from spherical aggregates (Figure 1A). Of note, actin can occur in MSCs either as g (globular)-actin or as f (filamentous)-actin, but phalloidin can only stain f-actin. Interestingly, mitochondrial mass followed the dynamics of f-actin. Lower quantities of mitochondria were found in adherent MSCs cultivated in HPL (Figure 1B).

### Stress Fiber Formation and Mitochondrial Dynamics

SF formation in response to HPL was investigated by Laser Scanning Microscopy in single 2D adherent P1 MSCs or MSCs that emanated from 3D spherical aggregates (Figures 1C,D). In order to scan single MSCs, 3D spherical MSC aggregates harboring 3,000 to 5,000 cells were adhered to glass slides and during a period of 16 h MSCs were allowed to emerge from the aggregate and remain adhered in the immediate surrounding eligible for scanning (Figure 1D). Here we can show, that MSCs grown in adherence to plastic surfaces formed thick and well-defined bundles of SFs compared to MSCs that emerged from spherical aggregates that showed only thin f-actin filaments (Figure 1C). Within the aggregates MSCs assembled only few SFs confirming previous results favoring aggregate cultures for stress reduced cultivation (Figure 1D). SFs are attached to the FA complexes, in which vinculin and paxillin are prominent proteins that are involved in facilitate the binding of FAs to integrins that bind to the underlying matrix. Here we can show that FAs behaved the same as SFs and remained expressed intensely after MSCs emerged from the spherical aggregates or when MSCs were cultivated in adherence to plastic surface (Figures 1C,D). The cultivation supplementation with HPL had no effect on the FA composition involving vinculin and paxillin under the conditions applied.

Mitochondrial distribution in single adherent MSCs showed that mitochondria were found in areas of high cytoplasmic activity and appeared condensed to rods and networks, according to fusion and fission dynamics (Figure 1C). MSCs that emerged from the spherical aggregates showed condensed mitochondria (Figure 1D) while MSCs in the cellular aggregate had a more punctuated morphology (data not shown). Interestingly, mitochondrial condensation did not appear perinuclear in adherent MSCs. The type II IF vimentin is important to support anchoring mitochondria at positions of high activity. Here we can show that vimentin distribution was higher in adherence to plastic surface, which goes hand in hand with the observations of the mitochondria distribution (Figures 1C,D).

### Influence of the Culture Media on the Occurrence of Stress Fiber Variants and Migratory Capability

**Figure 2:**
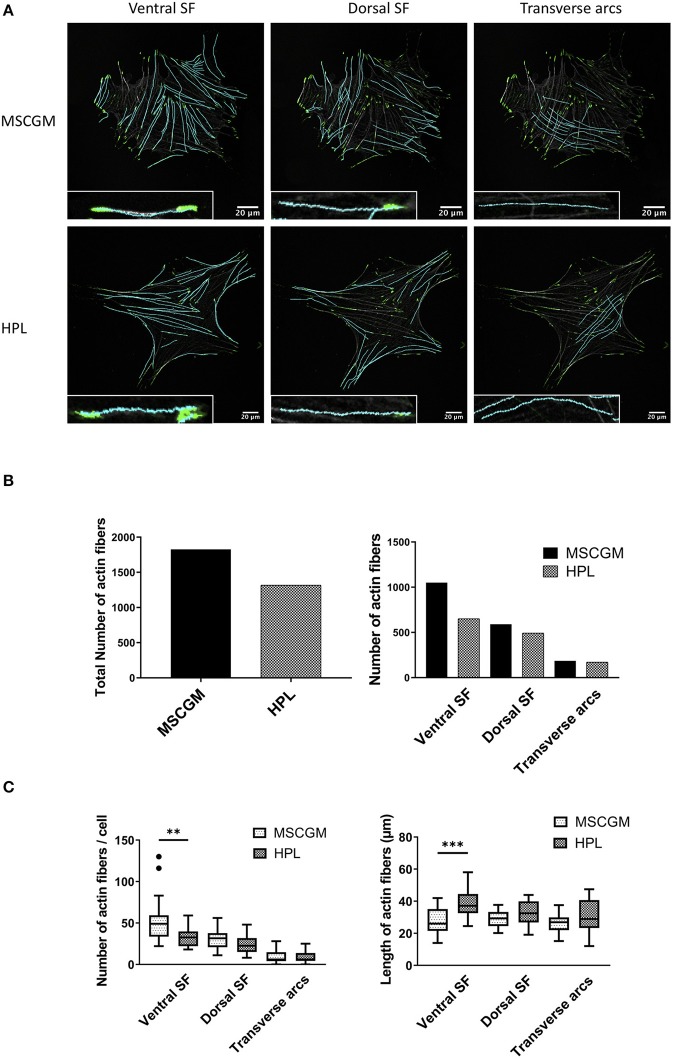
Examination of cytoskeleton morphology. **(A)** Images of ventral, dorsal stress fibers and transverse arcs compared to the influence of MSCGM™ or MSCBM™ with 8% HPL. Higher magnification of the individual fibers is shown in the lower left corner. **(B)** Left: Total numbers of stress fibers. Right: Total number of stress fibers split in ventral, dorsal stress fibers and transverse arcs. **(C)** Left: Number of fibers per cells, Mann-Whitney test. Right: Median of the length per cell, unpaired *t*-test. The same 20 cells per media were compared in **(B,C)**. **p < 0.01, ***p < 0.001.

Context-based analysis of confocal images allowed us the quantification of the SFs and the segregation of SFs in ventral SFs, dorsal SFs, and transverse arcs according to their connection with FAs (Figure 2A). Ventral SFs were segregated by their capability to anchor at both sides to FAs, dorsal SFs only bind one FA at one side, while the other side remained fixed to cytoplasmic organelles. Transverse arcs, in contrast do not bind to FAs at all but organize the tension within the cell. Here we can show an increase of the total number of SFs in thirty analyzed adherent P1 MSCs cultured in MSCGM™- medium (Figure 2B, left). This increase of actin fibers is comparable with the higher amount of actin determined in Figure 1A. Ventral SFs showed a response of cultivation where the number of ventral SFs decreased during cultivation of MSCs in HPL, while the length increased, dorsal SFs remained numerically constant. Transverse arcs, in contrast, remained constant both in number and length (Figures 2B,C). Perinuclear actin cap consists of SFs positioned above the nucleus that are mechanotransducers to convey force from the cell environment to the nucleus was not found in our system. When the migratory capability of P3 MSCs was investigated in a commercially available wound healing assay, where a 500 μm cell-free gap was introduced with a biocompatible silicone insert, we found an approximately 50% closure after 12 h and more than 90% after 24 h. No difference, however, was observed when P3 MSCs were cultivated either in MSCGM™- or in HPL supplemented medium (Supplementary Figures 3A–C).

### Topographic Imaging and Elasticity Measurements of MSCs Dependent on Culture Media

**Figure 3:**
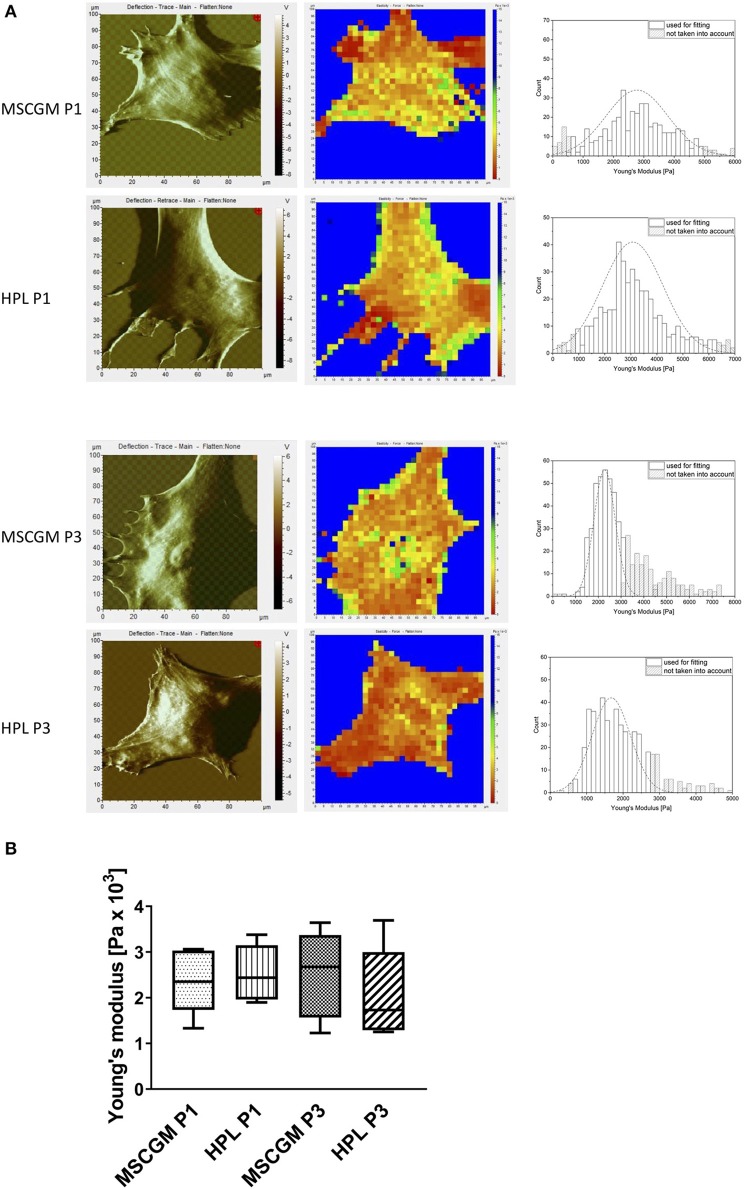
Examination of elasticity. **(A)** Left: Topographic images of membrane structures from MSCs cultured in MSCGM™ or MSCBM™ with 8% HPL from passage 1 and 3 determined by atomic force microscopy. Middle: Elasticity mapping. Right: Surface elasticity measurements given by the Young's moduli shown in a histogram. **(B)** Comparison of 1,024 single surface elasticity measurements from individual MSCs shown in a box plot diagram (*n* = 6).

Topographic images, to identify sub-membrane cytoskeletal structures in adherent P1 and P3 MSCs were performed by the AFM in deflection mode. MSCs showed a flat and spread morphology size (Figure 3A, left). Elasticity mapping and the calculation of Young's moduli of three MSCs of two batches were performed to investigate the effect of MSCGM™- medium or MSCBM™ with 8% HPL on the mechanical properties of P1 and P3 MSCs. Heat maps of 1,024 single measurements were carried out to show the stiffness or softness of the cultured MSCs (Figure 3A, middle). The individual data points were summarized in a histogram, the data points of the plastic substrate were excluded (Figure 3A, right). Young's moduli were presented in a boxplot for comparison to show that the change of the cellular compartment has no effect of the individual cell in influence of the media and passages on the elasticity and the mechanical characteristics (Figure 3B).

### Pattern Analysis of the Mitochondria

**Figure 4:**
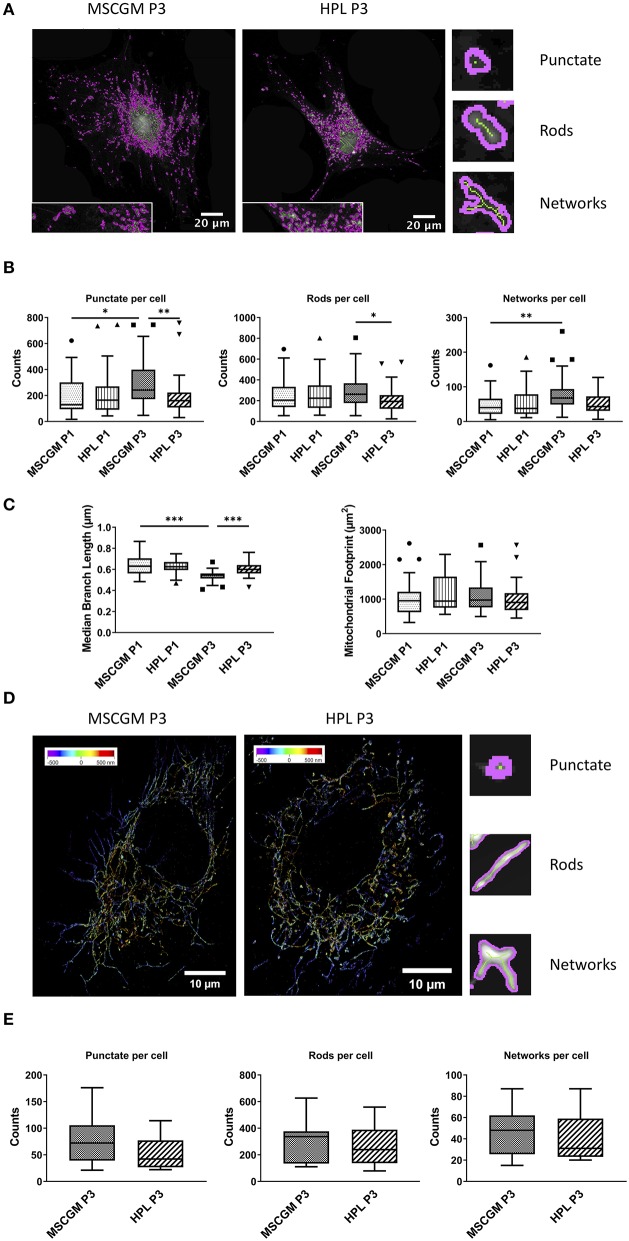
Mitochondrial pattern analysis and super-resolution imaging. **(A)** Images of Mitochondrial Network Analysis (MiNA) from passage 3 MSCs cultured in MSCGM™ or MSCBM™ with 8% HPL stained with MitoTracker™ **(B)** Top: Numbers of punctate mitochondrial organelles per cell compared in different media and from passage 1 and 3, Wilcoxon and Mann Whitney test. Middle: Numbers of mitochondrial rods per cell, Mann Whitney test. Bottom: Numbers of mitochondrial networks per cell, Wilcoxon test. MiNA image interpretations are shown next to the diagrams. **(C)** Left: Median branch length of the mitochondrial networks in μm per cell, paired and unpaired *t*-test. Right: Mitochondrial footprint in μm^2^ per cell. The same 30 cells per media or passage were compared in **(B,C)**. **(D)** Reconstructed fluorescence microscopy images (dSTORM) of mitochondria from passage 3 MSCs cultured in MSCGM™ or MSCBM™ with 8% HPL. **(E)** Numbers of punctate mitochondrial organelles as well as rods and networks per cell from passage 3 in different media. Data were calculated with Mitochondrial Network Analysis (MiNA), image interpretations are shown next to the diagrams. *p < 0.05, **p < 0.01, ***p < 0.001.

We used an ImageJ macro tool (Valente et al., 2017) to further analyze mitochondrial morphology in response to HPL in single adherent P1 or P3 MSCs. Here we could distinguish between unbranched punctate, rods and branched structures (networks) with high precision, the images are single P3 MSCs stained with MitoTracker™ analyzed with high resolution confocal microscopy (Figure 4A) in living cells. Unbranched punctate and networks were shown to be increased in adherent P3 MSCs compared to adherent P1 MSCs cultivated in MSCGM™- medium. Interestingly, when P3 adherent MSCs were cultured in HPL, the cells showed the same mitochondrial morphology as P1 MSCs (Figure 4B). When networks were further analyzed for branching (length of branched mitochondrial structures) we found the highest values in P1 adherent MSCs cultured in MSCGM™- medium (Figure 4C). Finally, mitochondrial rods were found to be decreased in adherent P3 MSCs cultivated in HPL (Figure 4B).

### Mitochondrial Imaging by dSTORM

MSCs were stained with an anti-mitochondria mAb, instead of MitoTracker™, to enable high resolution analysis of ATP5H mitochondrial synthase by a modified Olympus IX81 inverted epifluorescence microscope (Figure 4D). 3-D reconstructed high-resolution immunofluorescent images of 15 randomly selected single adherent permeabilized MSCs. When P3 adherent MSCs cultivated in MSCGM™- medium were compared with HPL and analyzed by the ImageJ macro tool, we found a trend toward a decrease in unbranched punctate mitochondria in P3. Interestingly, mitochondrial rods that count for the majority of mitochondrial structures and mitochondrial network structures showed no dependence on cultivation in HPL (Figure 4E).

### Determination of Immunological Molecules by Semi-quantitative RT-PCR

**Figure 5:**
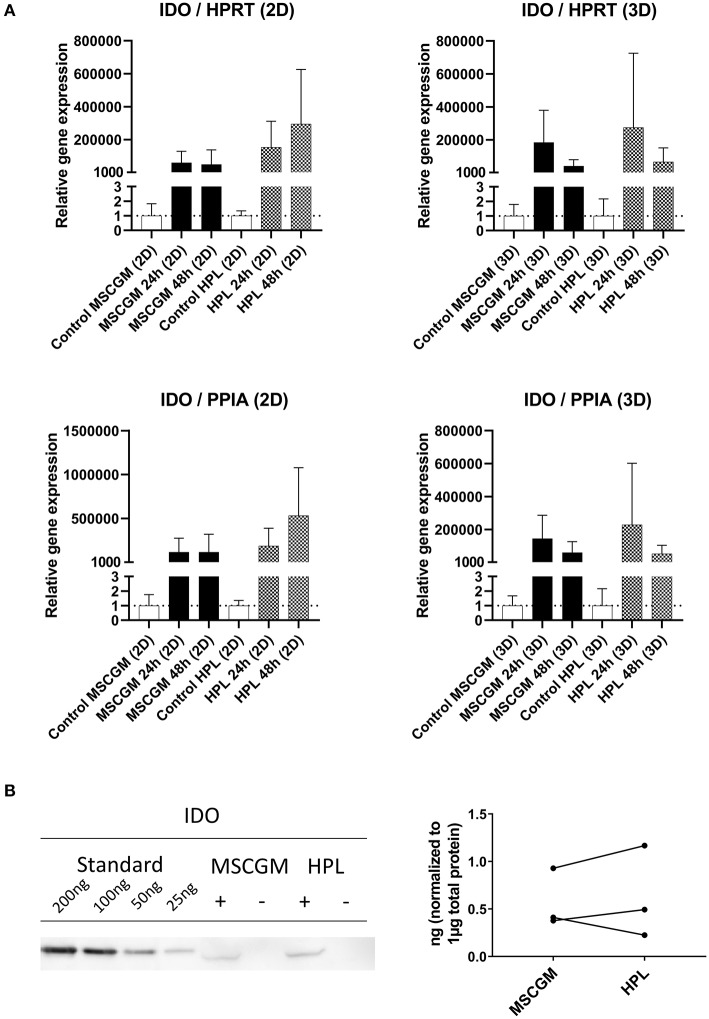
Expression of the immunological molecule IDO. **(A)** RT-PCR analysis of IDO gene expression in 2D and 3D MSCs for 24 and 48 h stimulation with IFN-γ/TNF-α cultured in MSCGM™ or MSCBM™ with 8% HPL and a control with unstimulated cells. Top: Relative gene expression levels of IDO normalized to the housekeeping gene HPRT. Bottom: Normalized to the housekeeping gene PPIA (2D *n* = 5; 3D *n* = 3). **(B)** Left: Western blot analysis of IDO protein expression stimulated with IFN-γ/TNF-α (+) or unstimulated (–) after 48 h. An internal standard of IDO ranging from 25 to 200 ng was included. One representative blot of three independent experiments is shown. Right: Comparison of IDO protein expression in ng per 1 μg total protein of three IFN-γ/TNF-α stimulated batches.

**Figure 6:**
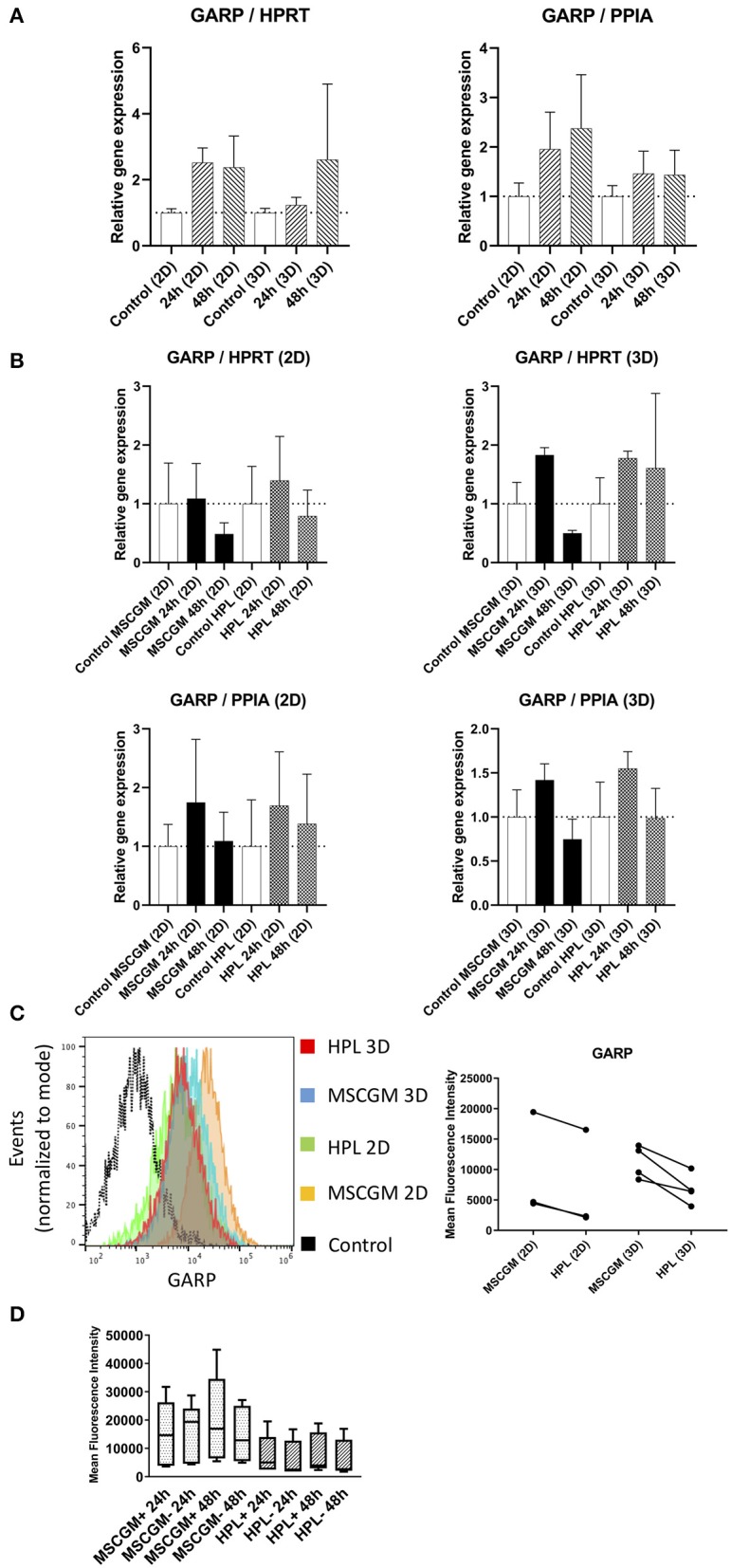
Expression of the immunological molecule GARP. **(A)** Relative gene expression levels of GARP after 24 and 48 h cultivation with MSCGM™ or MSCBM™ with 8% HPL and a control and a control at the beginning of cultivation time. Left: Normalized to the housekeeping gene HPRT (2D *n* = 5; 3D *n* = 3). Right: Normalized to the housekeeping gene PPIA (2D *n* = 5; 3D *n* = 3). **(B)** RT-PCR analysis of GARP gene expression in 2D and 3D MSCs for 24 and 48 h stimulation with IFN-γ/TNF-α cultured in MSCGM™ or MSCBM™ with 8% HPL and a control with unstimulated cells. Top: Normalized to the housekeeping gene HPRT. Bottom: Normalized to the housekeeping gene PPIA (2D *n* = 5; 3D *n* = 3). **(C)** Left: GARP measurements of living CD90^+^ MSCs in 2D vs. 3D cultured in different media determined by flow cytometry, 6,000 cells were compared. Right: Dot plot of the mean fluorescence intensity of three batches in 2D and four batches in 3D, 6,000 MSCs were investigated per batch. **(D)** Box plot of the mean fluorescence intensity of GARP protein expression of five batches unstimulated or stimulated with IFN-γ/TNF-α for 24 and 48 h.

Gene and protein expression of immunological molecules that mediate MSC functions and might rely directly on the status of SF formation and mitochondrial activity like GARP and IDO were analyzed before and after the stimulation with pro-inflammatory cytokines IFN-γ and TNF-α in 2D and 3D cultivated MSCs. Gene as well as IDO protein expression could only be found after stimulation with pro-inflammatory cytokines TNF-α and IFN-γ and not in unstimulated cultures. Due to high variance in IDO RT-PCR the normalization to two housekeeping genes HPRT and PPIA showed no increase in MSCs cultured in MSCBM™ with 8% HPL after 24 and 48 h of stimulation (Figure 5A). Protein expression of IDO analyzed by western blot after 48 h stimulation with IFN-γ and TNF-α showed an increase depending on HPL cultivation (Figure 5B) but this difference could only be found in two of three investigated batches. When gene expression of GARP was analyzed by RT-PCR in unstimulated adherent MSCs or MSCs from spherical aggregates, we found decreased GARP mRNA in cultures supplemented with HPL (Figure 6A). GARP protein followed gene expression (Figure 6C). Unlike IDO, GARP was constitutively expressed and not responsive to stimulation with IFN-γ and TNF-α, neither in 2D nor in 3D cultivated MSCs (Figures 6B,D), after 24 or 48 h.

## Discussion

Extensive research is needed to understand the mechanism how MSCs contribute to organ repair in order to rescue damaged tissue. After application, MSCs can (1) repair connective tissue disease or trauma by integration and differentiation into the required organ specific mature cells at the target site. Furthermore, (2) a locally released secretome of the therapeutic MSCs within the disease milieu can further lead to recovery of damaged organ cells. Finally, (3) a transfer of mitochondria from MSCs to damaged cells in the immediate surroundings by tunneling nanotubes can also facilitate regeneration. The distribution and direct impact of the three different mechanisms of MSC-mediated tissue repair is currently under debate as well as the efficacy of MSC-induced regeneration without post-transplant integration just by the local release of the MSCs' secretome and mitochondrial transfer. Here we investigate the importance of culture medium composition involving HPL as human-derived FBS alternative for MSC expansion. In addition, we investigate the impact of 2D cultivation in adherence to plastic surfaces and 3D cultivation in MSC spherical aggregates generated by hanging drop technology. We found that MSCs grown in adherence to plastic surfaces showed higher levels of f-actin protein and formed thick and well-defined bundles of SFs, while MSCs from spherical aggregates showed lower amounts of f-actin protein which correlated with thinner and more delicate f-actin filaments. The application of HPL led to a reduction in f-actin protein in MSCs grown in adherence to plastic surface while HPL had no effect in MSCs grown in spherical aggregates. It seems likely that HPL has a direct effect on 2D cultured MSC reducing actin stress fiber formation and mitochondrial mass due to the spectrum of cytokines, chemokines, and growth factors provided in the HPL preparation. These human derived bioactive factors were shown to be capable to modulate resiliency of cultured MSCs stressed with palmitate and could lower overall variance in MSC performance between donors (Boland et al., 2019). In adherent MSCs SFs are attached to the FA complex, in which vinculin and paxillin are prominent proteins facilitating the binding of FAs to integrins. Here we can show that FAs behaved the same as SFs and remained expressed intensely after MSCs emerged from the spherical aggregates or when MSCs were cultivated in adherence to the plastic surface. The cultivation supplementation HPL, in contrast, had no effect on FA composition involving vinculin and paxillin when MSCs are adherent to plastic surfaces to facilitate binding via integrins. When SFs were further segregated in ventral SFs, dorsal SFs and transverse arcs according to their anchorage to FAs, ventral SFs showed a response to cultivation where the number of ventral SFs decreased during cultivation of MSCs in HPL, while the length increased, dorsal SFs remained numerically constant. Transverse arcs, in contrast, remained constant both in number and length. This has an effect on the inner tension force of adherent MSCs and eventually on deformability as well as membrane elasticity. These results gave clear evidence for HPL-induced reduction of SF formation in MSCs adherent to the plastic surface, a recommended technology to expand MSCs *ex vivo* (*in vitro*) by the ISCT (Dominici et al., 2006). Interestingly, HPL had no effect when cells were cultivated in 3D spherical aggregates where cells have contact with their autologous matrix. We found a mix of collagen type I, fibronectin, vitronectin, collagen type IV deposition in spherical aggregates of MSCs after 7 days of cultivation (Rossmanith, 2018) and this autologous matrix can facilitate mechanobiological effects of the surrounding environment important for MSC fate determination (Zeiger et al., 2012; Yang et al., 2016). It was shown previously that SF formation can determine the membrane topography of MSCs where bold SFs induce an imprint on the covering cell membrane and the inner tension can indirectly contribute to changes in membrane elasticity of MSCs when measured by AFM (Burridge and Wittchen, 2013; Burridge and Guilluy, 2016). Here we can show that the membrane elasticity determined by the Young's modulus did not change upon the cultivation of adherent MSCs from P1 to P3 when 1024 single measurements were carried out. Due to high variance the decrease in membrane rigidity observed in adherent MSCs of P3 cultivated in the presence of HPL was not significant as compared to cultivation in MSCGM™- medium. Elasticity is a major biophysical property of a MSC and gives information on the resistance of the cell to deform under stress and can retune to its original shape when the strain is relieved. A relation between external force and force deformation exists (Carl and Schillers, 2008). When cell migration of adherent P3 MSCs was investigated in a commercially available wound healing assay no difference between P3 MSCs cultivated either in MSCGM™- or in HPL supplemented medium (Supplementary Figures 3A–C) could be observed. Taken together, these results indicate that an adaptation to cultivation conditions that can alter membrane elasticity and deformability of MSCs as well as their migratory capability could not be observed in adherent P3 MSCs.

Mitochondria are the powerhouses of the cell producing the majority of ATP through respiration and oxidative phosphorylation (OXPHOS) but besides energy generation, mitochondria also participate in calcium signaling, redox homeostasis and apoptosis, thereby mitochondria metabolism can predetermine the fate of transplanted MSCs. It was shown previously that mitochondria accumulation at sites within the cytoplasm of high-energy demand where local ATP production is essential for virtually all cellular functions. Mitochondrial fusion and fission dynamics that involves transportation by IFs is therefore an indicator of MSC fitness. Here we can show that the mitochondrial mass increased during cultivation of adherent MSCs from P1 to P3 as determined by flow cytometry and HPL culture supplementation prevented this increase. Mitochondria were either determined by MitoTracker™, a vital fluorescent lipophilic cationic dye, or by a specific mAb that determines ATP5H mitochondrial synthase and analyzed by 3D reconstructed high resolution immunofluorescence microscopy. We could distinguish between unbranched punctate, rods, and branched structures (networks) with high precision in living MSCs. Unbranched punctate and networks were shown to be increased in adherent P3 MSCs compared to adherent P1 MSCs cultivated in MSCGM™- medium. When P3 adherent MSCs were cultured in HPL, cells showed the same mitochondrial morphology as P1 MSCs. When networks were analyzed for length of branched mitochondrial structures, we found the highest values in P1 adherent MSCs cultured in MSCGM™- medium. When P3 adherent MSCs cultivated in MSCGM™- medium were analyzed by dSTORM and calculated by ImageJ we found a trend toward a decrease in unbranched punctate mitochondria in P3 adherent cultivated in HPL, confirming previous experiments using the MitoTracker™. Interestingly, mitochondrial rods that count for the majority of mitochondrial structures found and mitochondrial network structures that appear as physically interconnected networks showed no dependence on cultivation in HPL when analyzed by dSTORM. dSTORM has the potential to examine mitochondrial reconstruction and semi-automated analysis of immunofluorescent images with high fidelity. It was shown previously that cultivation-induced mitochondrial remodeling involves mitochondrial fission that plays a crucial role in the segregation of aberrant mitochondrial fragments from the remaining tubular network as well as mitophagy. Mitochondria fission is regulated by cytoplasmic proteins Drp1 and Fis1 localized on the outer membrane of mitochondria, while NIX, BNIP3, FUNDC1, PINK (PTEN-induced putative kinase), and PARK2 (E3-ubiquitin ligase Parkin) dependent mechanisms are involved in determining damaged mitochondria and forward them for mitophagy (Bragoszewski et al., 2017; Bravo-San Pedro et al., 2017). Mitochondrial fusion, on the other hand, is induced by mitofusins (Mfn)1 and Mfn2 as well as optic atrophy protein (Opa)1. These outer mitochondrial membrane proteins mediate adhesion and fusion through their cytoplasmic exposed GTPase domain. This fusion mechanism promotes the formation of long tubular structured mitochondria with further interconnection into reticular structures as seen in adherent MSCs in P3. The deterioration of mitochondrial appearance in P3 MSCs was accompanied by nuclear budding as observed by DAPI-staining. A major component of maintaining cellular homeostasis is the recognition and removal of dysfunctional mitochondria, as mitochondria are an essential source of ATP for cellular function. We could further show, that MSCs grown in spherical hanging droplet cultures omitting adherence, in contrast, showed a mitochondrial network evenly distributed throughout the entire cytoplasm, indicating that this cultivation technique induced the least stress to the cultured MSCs.

MSCs facilitate immunomodulatory capabilities involving a network of regulatory pathways. These pathways need to be orchestrated both spatially and chronologically and rely on a dynamic cytoskeleton as well as energy in order to function properly, but the precise mechanism and the regulatory molecules involved are still an area of debate. Here we have selected two prominent immunomodulatory molecules indoleamine-2,3-dioxygenase (IDO), a tryptophan-catabolizing heme-containing enzyme that facilitates a rapid consumption of tryptophan from the local microenvironment and glycoprotein A repetitions predominant (GARP) a membrane receptor binding latency-associated peptide (LAP)/TGF-β1 to study their dependence on cultivation in 2D or 3D and the effect of HPL. IDO is of particular importance because removal of L-tryptophan by IDO starves the environment of this essential amino acid and stress-response pathways such as GCN2 and mTOR signaling pathways are sensitive to amino-acid withdrawal. The biologically active tryptophan catabolites such as kynurenine metabolites can contribute to a *de novo* induction of FoxP3. MSCs do not express IDO constitutively, but IDO can be induced by several factors, among which IFN-γ plays a prominent role (Ling et al., 2014; Rožman and Švajger, 2018). Here we can show that gene expression of IDO in MSCs was only found after stimulation with pro-inflammatory cytokines TNF-α and IFN-γ as IDO was not expressed in unstimulated MSCs. MSCs cultured with HPL supplementation showed no further increase of IDO mRNA due to high variance. IDO protein expression was found only by western blotting, because IDO could not be investigated by flow cytometry because it remained cytoplasmic. An increase in protein expression of IDO in two of three batches analyzed by western blotting could be observed after 48 h of stimulation with TNF-α and IFN-γ when cultured in HPL compared to MSCGM™- medium. The second molecule GARP that was shown previously to be expressed by MSCs, activated FoxP3^+^ Tregs, and megakaryocytes/platelets co-localized with TGF-β1, thereby controlling immune regulatory molecules such as FoxP3 (Carrillo-Galvez et al., 2015). Unlike IDO, GARP was constitutively expressed and was found independent of stimulation with TNF-α and IFN-γ. Constitutive GARP expression on the surface of MSCs did not change when HPL was used as medium supplement.

In conclusion, we can say that cultivation in 3D is beneficial for MSCs to maintain low SF formation and physiological membrane elasticity as well as ensure adequate mitochondrial function and ATP supply. Cultivation in 2D, where MSCs adhere to the plastic surface, led to the intensive ventral SF formation where bold f-actin bundles span the entire length of the MSC and perinuclear mitochondrial accumulation. Ventral SFs anchored at two sides to FA can set up an internal force of contraction which enhances membrane elasticity. Serum substitution with HPL could partially reverse this effect in 2D cultivated MSCs leading to lower levels of f-actin protein and mitochondrial mass. For a successful therapeutic application of MSCs in a clinical setting, clinicians need to understand the complex regulatory pathways influencing MSC cyto-architecture, since generation of inner forces induced by ventral SF formation has an effect on membrane elasticity, deformability, and ability of MSCs to migrate. These physical properties of MSCs combined with mitochondrial dynamics can determine the immunomodulatory function of MSCs. Here we can show that IDO was not expressed in unstimulated MSCs and relayed on stimulation with IFN-γ in both 2D and 3D MSC cultures. The immune regulatory molecule GARP was found to be constitutively expressed independent of 2D or 3D cultivation with no additional upregulation upon stimulation with IFN-γ and TNF-α. Interestingly, HPL had no effect on IDO or GARP expression in our 2D or 3D MSC culture systems. This knowledge is relevant because MSCs, upon administration, can migrate to sites of injury, eventually engraft and differentiate into functional mature organ cells, but definitively secrete cytokines, chemokines, hormones and growth factors as well as extracellular vesicles at the target site. To perform combined investigations on SF formation, mitochondrial morphology and deformability of the MSC membrane and link these results with functional properties like the expression of immunomodulatory molecules is important prior to administration and a prerequisite to translate these results in a humanized animal model in the future.

## Data Availability Statement

The datasets generated for this study are available on request to the corresponding author.

## Ethics Statement

The studies involving human participants were reviewed and approved by EK791/2008, EK1192/2015, GS1-EK-4/312-2015. The patients/participants provided their written informed consent to participate in this study.

## Author Contributions

MF conceived the presented idea and supervised the project as principle investigator. MP conducted the experiments and was responsible for acquisition, analysis, and interpretation of data for the work. MP drafted and MF contributed to the final version of the manuscript. VW revised the draft critically for important intellectual content. ER contributed at cell isolation, acquisition, and interpretation of data. CM and AE assisted with the atomic force microscopy and helped carry out the analysis. FH and JJ contributed the 3D dSTORM analysis.

### Conflict of Interest

The authors declare that the research was conducted in the absence of any commercial or financial relationships that could be construed as a potential conflict of interest.
