# Supplementary material for: Influence of Platelet Lysate on 2D and 3D Amniotic Mesenchymal Stem Cell Cultures
Source: Front Bioeng Biotechnol. 2019 Nov 15;7:338. doi: 10.3389/fbioe.2019.00338 (PMC6873824; doi:10.3389/fbioe.2019.00338)
Supplement: Supplementary file 1 [file Table_1.DOCX]

Supplementary Material

# Supplementary Figures


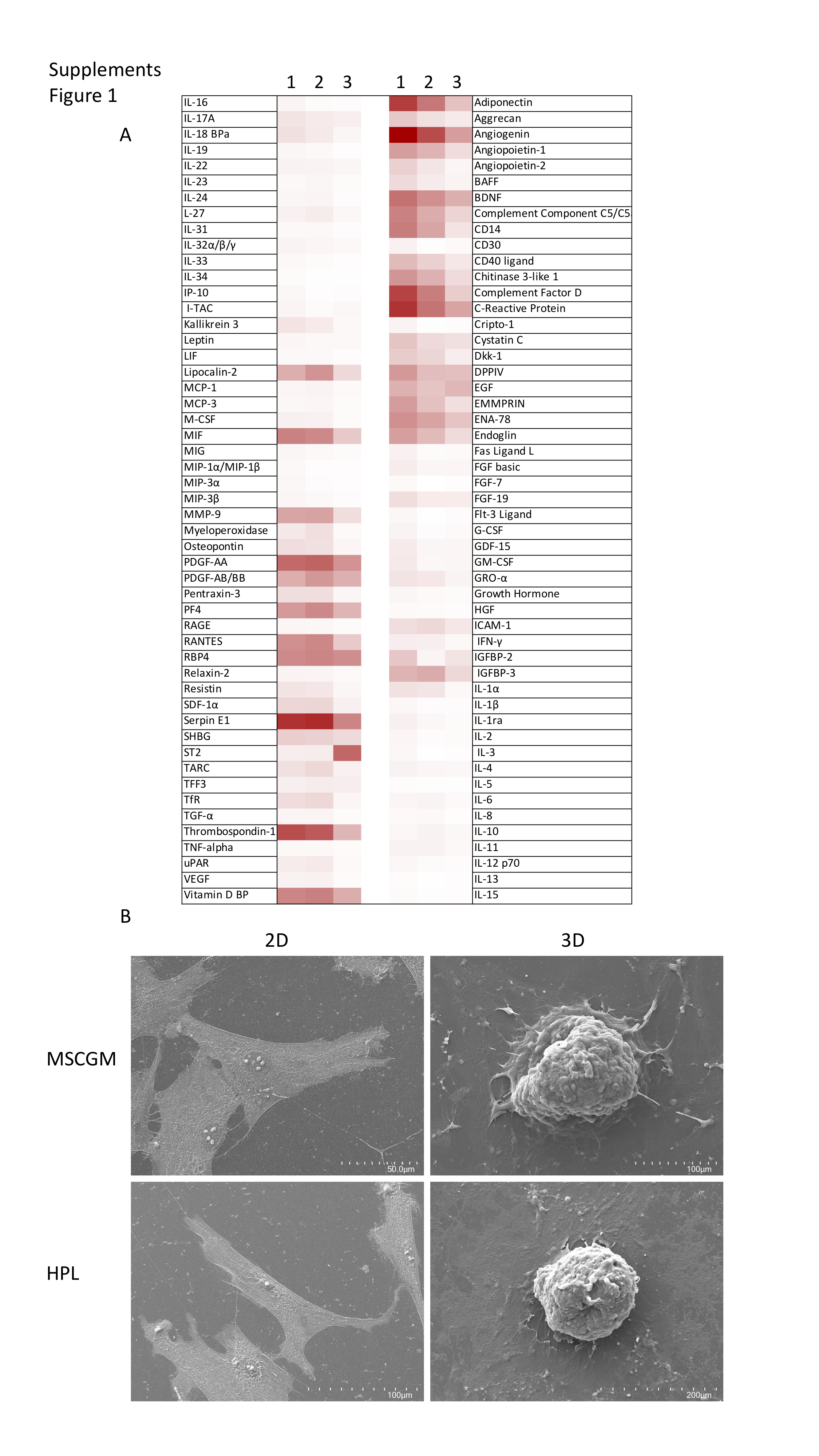


**Supplementary Figure 1. Platelet Lysate composition and morphology of MSCs.**

**(A)** Heat map of three different platelet lysate batches analyzed with a human cytokine array, dark red indicates a high expression. **(B)** Images of MSC spheroids as well as single adherent MSCs cultured in MSCGM^TM^ or MSCBM^TM^ with 8 % HPL generated by scanning electron microscopy.


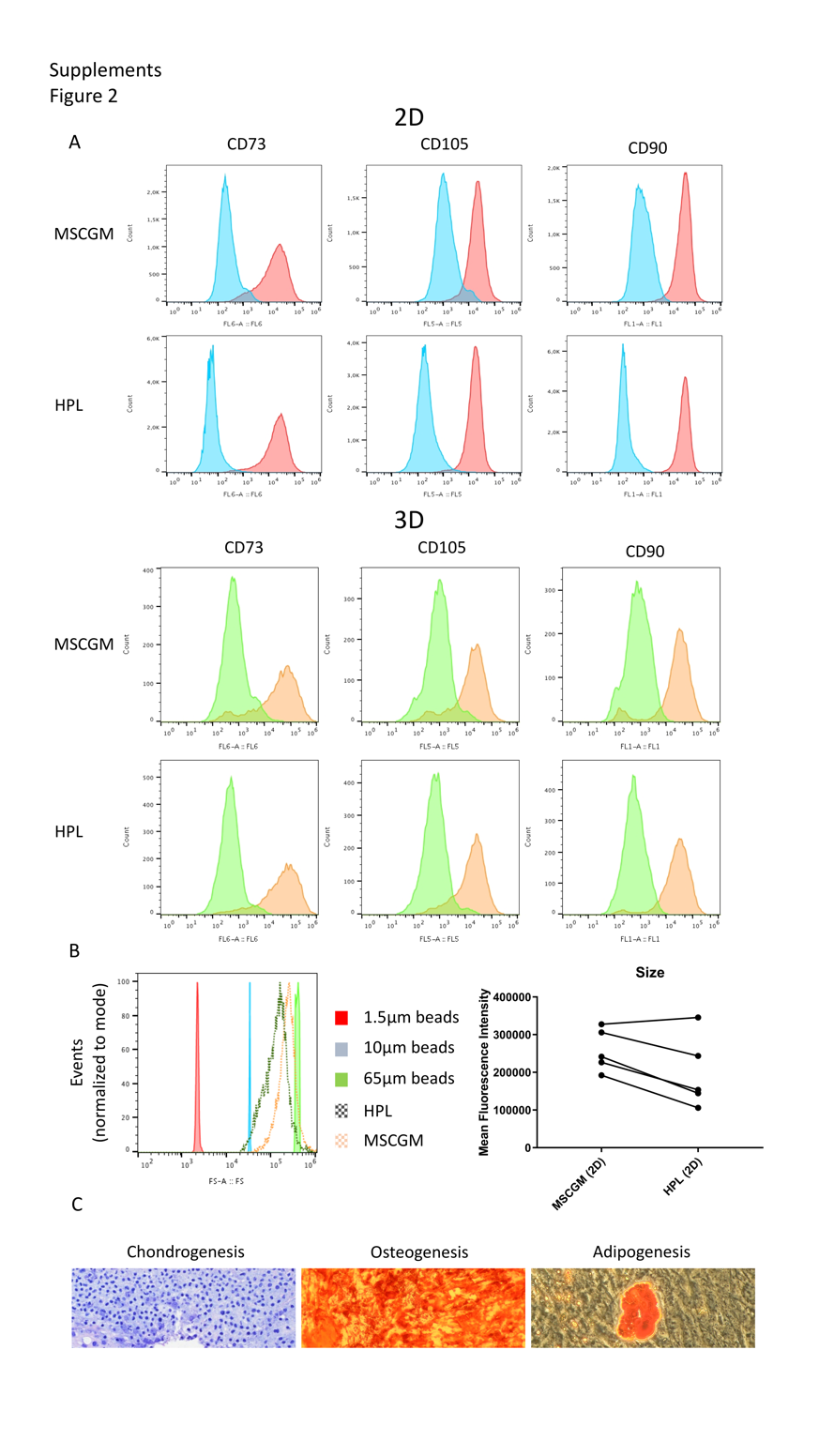


**Supplementary Figure 2. Phenotyping and size measurements of MSCs.**

**(A)** Flow cytometric measurements of living 2D vs 3D MSCs cultured in MSCGM^TM^ or MSCBM^TM^ with 8 % HPL stained with anti-CD73, anti-CD90 and anti-CD105. **(B)** Size measurements of living 2D MSCs grown in different media by flow cytometry using 1.5 µm, 10 µm and 65 µm silica beads. Diagram compares the mean fluorescence intensity of living MSCs. **(C)** Three-lineage differentiation **Left:** Alcian blue staining indicates chondrogenic differentiation. **Middle:** Alizarin red staining indicates osteogenic differentiation. **Right:** Oil red staining indicates adipogenic differentiation.


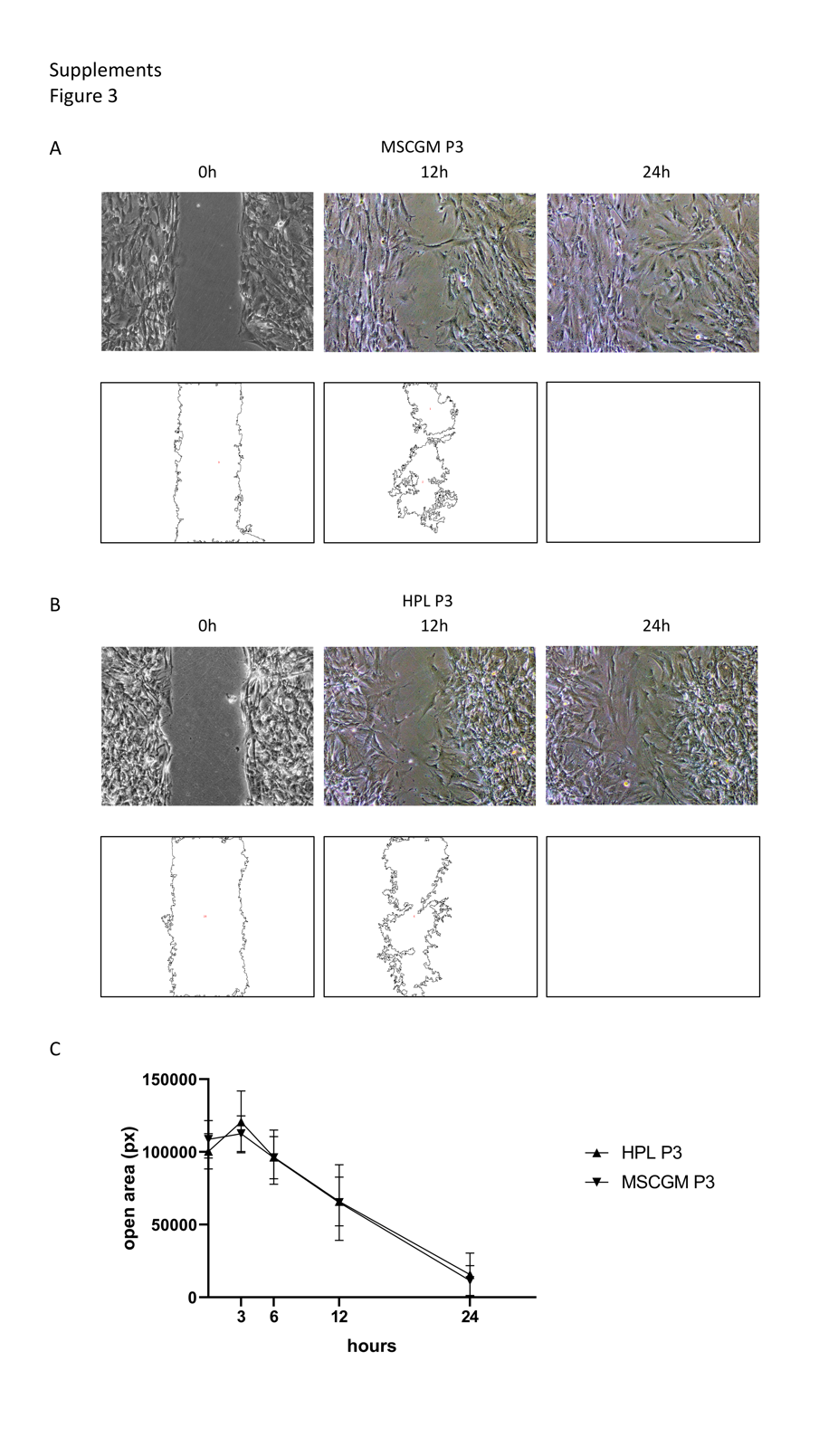


**Supplementary Figure 3. Migration assay**

**(A) Top:** Phase contrast images from MSCs cultured in MSCGM^TM^ from passage 3 at timepoint 0h, 12h and 24h, magnification was 4x (n=5) **Bottom:** Image analysis with FIJI **(B) Top:** Phase contrast images from MSCs cultured in MSCBM^TM^ with 8 % HPL from passage 3 at timepoint 0h, 12h and 24h, magnification was 4x (n=5) **Bottom:** Image analysis with FIJI **(C)** Comparison of the closure time course of adherent MSCs from passage 3 cultured in MSCGM^TM^ or MSCBM^TM^ with 8 % HPL
